# Protease-Activated Receptor 2 Promotes Pro-Atherogenic Effects through Transactivation of the VEGF Receptor 2 in Human Vascular Smooth Muscle Cells

**DOI:** 10.3389/fphar.2016.00497

**Published:** 2017-01-04

**Authors:** Ira Indrakusuma, Tania Romacho, Jürgen Eckel

**Affiliations:** ^1^Paul-Langerhans-Group for Integrative Physiology, German Diabetes CenterDüsseldorf, Germany; ^2^German Center for Diabetes Research (DZD e.V.)Düsseldorf, Germany

**Keywords:** obesity, PAR2, VEGFR2, smooth muscle cell proliferation, atherosclerosis

## Abstract

**Background:** Obesity is associated with impaired vascular function. In the cardiovascular system, protease-activated receptor 2 (PAR2) exerts multiple functions such as the control of the vascular tone. In pathological conditions, PAR2 is related to vascular inflammation. However, little is known about the impact of obesity on PAR2 in the vasculature. Therefore, we explored the role of PAR2 as a potential link between obesity and cardiovascular diseases.

**Methods:** C57BL/6 mice were fed with either a chow or a 60% high fat diet for 24 weeks prior to isolation of aortas. Furthermore, human coronary artery endothelial cells (HCAEC) and human coronary smooth muscle cells (HCSMC) were treated with conditioned medium obtained from *in vitro* differentiated primary human adipocytes. To investigate receptor interaction vascular endothelial growth factor receptor 2 (VEGFR2) was blocked by exposure to calcium dobesilate and a VEGFR2 neutralization antibody, before treatment with PAR2 activating peptide. Student's *t*-test or one-way were used to determine statistical significance.

**Results:** Both, high fat diet and exposure to conditioned medium increased PAR2 expression in aortas and human vascular cells, respectively. In HCSMC, conditioned medium elicited proliferation as well as cyclooxygenase 2 induction, which was suppressed by the PAR2 antagonist GB83. Specific activation of PAR2 by the PAR2 activating peptide induced proliferation and cyclooxygenase 2 expression which were abolished by blocking the VEGFR2. Additionally, treatment of HCSMC with the PAR2 activating peptide triggered VEGFR2 phosphorylation.

**Conclusion:** Under obesogenic conditions, where circulating levels of pro-inflammatory adipokines are elevated, PAR2 arises as an important player linking obesity-related adipose tissue inflammation to atherogenesis. We show for the first time that the underlying mechanisms of these pro-atherogenic effects involve a potential transactivation of the VEGFR2 by PAR2.

**Graphical Abstract:**
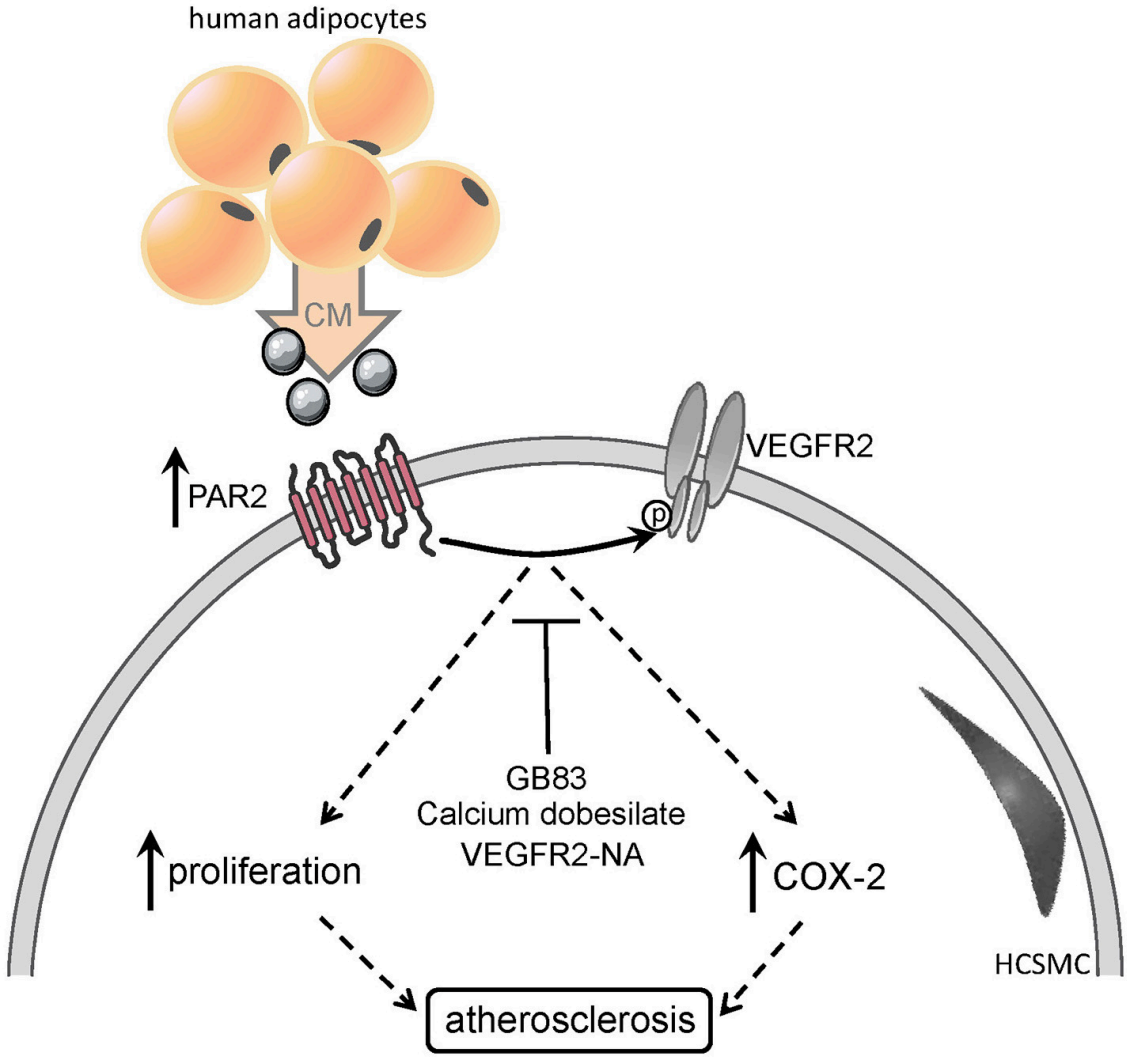
**Upregulation and activation of PAR2 by elevated levels of pro-inflammatory cytokines, lead to pro- atherogenic events, such as smooth muscle cell proliferation, COX2 and NFkB induction**.

## Introduction

Obesity is associated with comorbidities such as type 2 diabetes and cardiovascular diseases (CVD) (Despres, 2012). CVD are the main cause for deaths in diabetes mellitus (Morrish et al., 2001) and lead to a significant increase in obesity-associated mortality (Lloyd-Jones et al., 2010). The development of obesity is a consequence of imbalance between energy intake and energy expenditure causing storage of excessive energy in the adipose tissue (AT) (Hill et al., 2012). Besides being a storage organ, it is well established that AT influences systemic metabolism as an endocrine organ secreting a variety of proteins referred to as adipokines (Trayhurn et al., 2011). During the progress of obesity AT undergoes modulation such as the enlargement of adipocytes, or a switch in the secretome toward a prominent release of pro-inflammatory adipokines (Ouchi et al., 2011). In turn, these adipokines are known as inflammatory factors promoting insulin resistance (Hotamisligil et al., 1995). Furthermore, it has been proposed that high levels of circulating pro-inflammatory adipokines found in diseases such as obesity and type 2 diabetes mellitus have an impact on cardiovascular function (Greenberg and Obin, 2006). Thus, adipokines represent a molecular link between metabolic and cardiovascular diseases (Taube et al., 2012). There is evidence that they can directly exert deleterious effects on both endothelial and smooth muscle cells resulting in damage of the vasculature (Karastergiou and Mohamed-Ali, 2010; Lamers et al., 2011; Schlich et al., 2013).

In 1994, protease-activated receptor 2 (PAR2), a seven transmembrane domain, G-protein-coupled receptor, was discovered by Nystedt et al. (1994) and has been found to be expressed in endothelial (Mirza et al., 1996) as well as in smooth muscle cells (Molino et al., 1998). While PAR1, PAR3, and PAR4 activation is driven by thrombin, PAR2 is activated by serine proteases such as tryptase, factor Xa, trypsin and the TF-FVIIa complex (Adams et al., 2011). The proteolytic cleavage of the extracellular N-terminal domain leads to the unmasking of a tethered ligand which then binds to a binding pocket in the receptor (Dery et al., 1998). In addition, PAR2 activation can be triggered by synthetic peptides mimicking the sequence of the tethered ligand (Macfarlane et al., 2001). In the vascular wall, activation of PAR2 initiates multiple signaling pathways exerting distinct responses, among others the control of vascular tone, and coagulation (Sriwai et al., 2013; Zhao et al., 2014).

Under pathological conditions, PAR2 is involved in cardiovascular responses such as vasorelaxation and vasoconstriction, as well as inflammatory processes (Hirano and Kanaide, 2003; Aman et al., 2010; Adams et al., 2011). For instance, administration of PAR2 agonists such as SLIGKV or trypsin induce proliferation in smooth muscle (Bono et al., 1997) and endothelial cells (Mirza et al., 1996). Moreover, smooth muscle cell proliferation via PAR2 can be triggered by pro-inflammatory adipokines such as DPP4 (Wronkowitz et al., 2014). *In vivo*, PAR2 activation drives the development of hypertension (Emilsson et al., 1997; Cicala et al., 1999) and PAR2 expression is enhanced in aortas of diabetic mice and in human coronary atherosclerotic lesions (McGuire, 2004; Napoli et al., 2004). Furthermore, upregulation of PAR2 content also occurs in endothelial cells and coronary arteries after treatment with inflammatory stimuli such as interleukin (IL)-1α, IL-1β, and tumor necrosis factor (TNF)-α (Nystedt et al., 1996; Hamilton et al., 2001; Ritchie et al., 2007). Overall, these studies suggest that PAR2 is an important player in both endothelial and smooth muscle cell function as well as in the overall control of vascular reactivity. However, the role of PAR2 in obesity-related vascular diseases remains unclear. Therefore, we addressed the importance of PAR2 in the vasculature under obesogenic conditions.

## Materials and methods

### Animal model

Animal care and use were approved by the local Animal Ethics Committee according to the principles outlined in the European Commission Council Directive (86/609/EEC). C57BL/6J mice were obtained from Jackson laboratories. Twelve week old mice were either fed a high fat diet (HFD, 60% kcal fat; Research diets D12492) or standard chow diet over 24 weeks. Animals were housed in polyacrylamide cages under temperature control (22°C) and a 12 h light/dark cycle. Mice were weighted every 4 weeks. After 24 weeks on the respective diets mice were sacrificed by cervical dislocation. Aortas were collected, cleaned of fat, snap frozen in liquid nitrogen and stored at −80°C until further analysis.

### Preparation of explants

Visceral AT from chow and HFD animals were collected in cold PBS supplemented with an antibiotic-antimycotic solution (Invitrogen, Carlsbad, CA, USA). Connective tissue and vessels were removed and fat pads were cut into small pieces. After washing three times in PBS, liquid was removed by putting explants on a sterile mesh. Relatively dry fat pieces were weighted and 100 mg of AT were incubated in 1 ml of HCSMC starvation medium (fetal calf serum (FCS)-free DMEM low glucose (Invitrogen, Carlsbad, CA, USA) at 37°C and 5%CO_2_ to generate conditioned medium (CM). After 24 h CM was collected and centrifuged at 1200 rpm for 10 min and stored at −20°C until further use.

### Isolation of preadipocytes and generation of human adipocyte CM

Preadipocytes were isolated from human abdominal AT obtained from moderately overweight or obese subjects undergoing plastic surgery following the protocol previously described and being approved by the ethical committee of the Heinrich-Heine-University (Düsseldorf, Germany; Hauner et al., 1995).

Preadipocytes were cultured in DMEM/F12 medium (Gibco Invitrogen, Carlsbad, CA, USA) supplemented with 33 μmol/l biotin (Sigma-Aldrich, Schnelldorf, Germany), 17 μmol/l d-panthothenic-acid (Sigma-Aldrich, Schnelldorf, Germany), 14 nmol/l NaHCO_3_ (Merck, Darmstadt, Germany), human insulin (100 nM, Sigma-Aldrich, Schnelldorf, Germany), dexamethasone (1 μM, Sigma-Aldrich, Schnelldorf, Germany), and 10% FCS (Gibco Invitrogen, Carlsbad, CA, USA) and grown until 90% confluence. Then differentiation was induced by addition of 0.25 μM troglitazone (Sigma-Aldrich, Munich, Germany) and 0.2 mM IBMX (Sigma-Aldrich, Munich, Germany). The switch to differentiation medium is indicated as day 0. After 7 days, medium was changed to maintenance medium (differentiation medium deprived of troglitazone and IBMX) until day 14. Medium was changed every 2–3 days. On day 14, cells were treated with either human coronary artery endothelial cell (HCAEC) starvation medium composed of endothelial cell basal medium MV and 5% FCS or HCSMC starvation medium (DMEM low glucose, 0% FCS) for 48 h. CM was collected, centrifuged at 1200 rpm for 10 min and stored at −20°C until further use.

### Cell culture

HCAEC obtained from three different donors were purchased from PromoCell (Heidelberg, Germany) and cultured in endothelial cell basal medium MV (PromoCell, Heidelberg, Germany) supplemented with 20% FCS, 1 μg/ml hydrocortisone, 0.004 ml/ml ECGS and 10 ng/ml EGF at 37° C and 5% CO_2_. Cells between passages 4–7 were used for experiments. When 90% confluence was reached HCAEC were washed with PBS and treated with CM. HCSMC from four different donors were purchased from PromoCell (Heidelberg, Germany), tebu-bio (Offenbach, Germany), and Lonza (Basel, Switzerland) were seeded in HCSMC growth medium (DMEM low glucose, Invitrogen, Carlsbad, CA, USA) containing 10% FCS and cultivated according to manufacturer's protocol at 37° C and 5% CO_2_. After reaching 90% confluence, cells were washed with PBS and serum starved for 24 h. HCSMC were then treated with CM or PAR2 activating peptide (PAR2-AP, SLIGKV-NH_2_; 50 μM, Bachem, Bubendorf, Switzerland). All experiments were performed in cells at passage 3.

### Immunoblotting

Cells were lysed in a lysis buffer composed of 50 mM HEPES (Promocell, Heidelberg, Germany), 1% TritonX100 (Sigma-Aldrich, Munich, Germany), complete protease inhibitor and PhosStop phosphatase inhibitor cocktail at pH 7.4 (Roche, Basel Switzerland). Protein lysates were diluted with Laemmli buffer and denatured for 5 min at 94°C. Five microgram protein were loaded on a 10% gel SDS-PAGE and transferred to a PVDF membrane during blotting process. Reagents for SDS-PAGE were supplied by Amersham Pharmacia Biotech (Braunschweig, Germany) and by Sigma-Aldrich (Munich, Germany). Blots were blocked in a Tris-buffered saline solution containing 0.1% Tween and 5% milk powder and incubated with antibodies against: PAR2 (SAM11: sc-13504, Santa Cruz Biotechnology, Heidelberg, Germany), COX2 (#4842), GAPDH (#2118), phospho-VEGFR2 (Tyr1059) (#3817), VEGFR2 (#2479), NF-κB p65 (#8242), and phospho-NF-κB p65 (Ser536) (#3033). Unless otherwise stated, all antibodies were purchased from Cell signaling Technology (Frankfurt, Germany). We used the corresponding secondary HRP-coupled antibodies against mouse and rabbit (Promega, Mannheim, Germany). After washing, blots were exposed to Immobilon HRP substrate (Millipore, Billerica, MA, USA) and analyzed with a VersaDoc 4000 MP work station (BIO-RAD, Munich, Germany).

### qRT-PCR

RNA isolation of human cells was performed in RLT lysis buffer working solution containing 1% β-mercaptoethanol according to manufacturer's protocols. Murine aortas were lysed in 1 ml Tripure (Roche, Mannheim, Germany) by a tissuelyser II (Qiagen, Hilden, Germany) for 5 min at 29 s^−1^ for mRNA isolation. mRNA purification of cells and aortas was done with an RNeasy Kit (Qiagen, Hilden, Germany) and content of mRNA was measured with a NanoDrop2000 (Thermo Scientific, Schwerte, Germany). All samples were reversely transcribed into 1 μg/μl of cDNA using an Omniscript RT Kit (Qiagen, Venlo, Netherlands) and mRNA levels were determined by StepOne Plus real-time PCR system (Applied Biosystems, Carlsbad, CA, USA). Primers from Qiagen were used: Hs_F2RL1_1_SG (QT02589377), Hs_ACTB_2_SG (QT01680476) Hs_PTGS2_SG (QF00461055), mM_F2RL1 (QT02255330), and Mm_Rn18S (QT02448075).

### Proliferation assay

Prior to determination of proliferation, HCSMC were starved for 24 h. Cells were pre-incubated with the specific PAR2 antagonist GB83 (10 μM, Axon Medchem, dissolved in DMSO), the vascular endothelial growth factor receptor 2 neutralization antibody (VEGFR2-NA, R&D Systems Wiesbaden-Nordenstadt, Germany, MAB3572) or calcium dobesilate (Sigma-Aldrich, Schnelldorf, Germany) for 1 h. Subsequently, cells were treated either with CM alone, CM in combination with GB83, the PAR2 activating peptide (PAR2-AP, SLIGKV-NH_2_; 50 μM, Bachem, Bubendorf, Switzerland), or PAR-AP in combination with the VEGFR2-NA or dobesilate for 24 h. Five percentage FCS was used as a positive control. All treatments contained 10% BrdU. Proliferation was assessed by measuring BrdU incorporation (Proliferation Assay, Roche, Mannheim, Germany) with a microplate reader (Infinite M200, Tecan GmbH, Männersdorf, Switzerland).

### Caspase 3/7 activity assay

HCAEC were seeded in a 96-well plate and cultured for 24 h. Cells were pre-treated with GB83 for 1 h and treated with CM alone or in combination with GB83 (10 μM) for 18 h. H_2_O_2_ (200 μM, Sigma-Aldrich, Schnelldorf, Germany) was used as a positive control. Caspase 3/7 activity was measured by a Caspase-Glo® 3/7 Assay (Promega, Mannheim, Germany) as described in the manufacturer's protocol. Luminescence was measured in a microplate reader.

### VEGF release

In order to monitor vascular endothelial growth factor (VEGF) release from HCSMC, supernatants were collected, centrifuged, and stored at −80°C. Supernatants were analyzed with a VEGF ELISA Kit purchased from R&D Systems (Wiesbaden-Nordenstadt, Germany).

### Adipokine array

The secretion profile of murine adipose explants was analyzed by usage of a proteome profiler (R&D Systems, Wiesbaden-Nordenstadt, Germany) according to manufacturer's protocol. Membranes were incubated with murine CM from chow and HFD-fed animals.

### Statistical analysis

Statistical analysis was performed using the GraphPad Prism software (La Jolla, CA, USA). Unpaired two-tailed Student's *t*-test or one-way ANOVA (*post hoc* test: Bonferroni or Dunnett's) were used to determine statistical significance considering a *p*-value below 0.05 as statistically significant. Data are expressed as mean values ± SEM.

## Results

### PAR2 expression is elevated by HFD in murine aortas and by human adipocyte-derived factors in HCAEC and HCSMC

**Figure 1:**
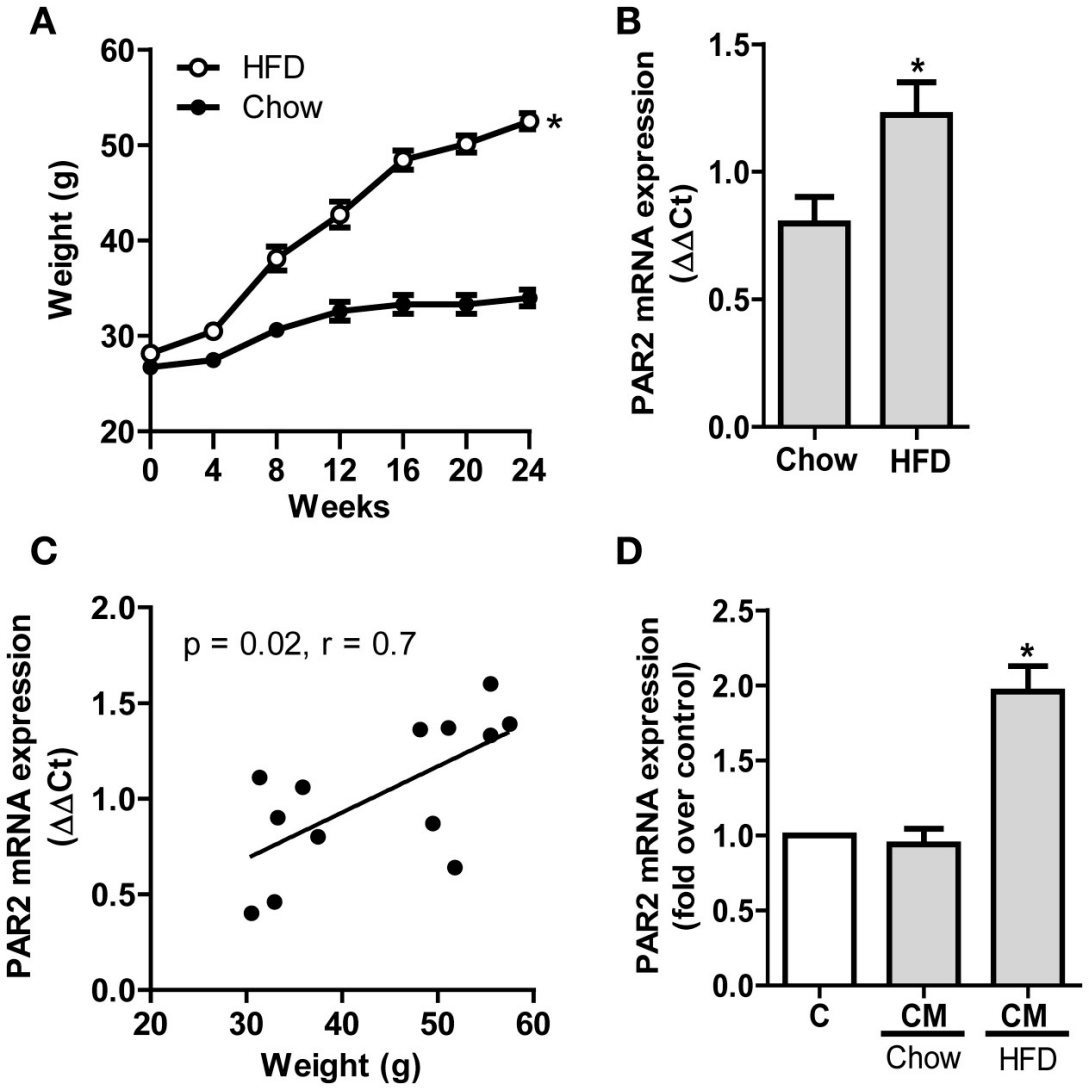
**HFD induces PAR2 expression in the vascular wall. (A)** Weight gain in C57BL/6J wild type mice under HFD or chow diet for 24 weeks; *n* = 11–27. **(B)** PAR2 mRNA expression in murine aortas after 24 weeks. PAR2 expression was normalized to 18S mRNA levels; *n* = 7. **(C)** Correlation of PAR2 mRNA expression in murine aortas and weight of respective animals; *n* = 13. **(D)** CM from murine epidydimal AT of chow- and HFD-fed mice were used to determine induction of PAR2 mRNA in HCSMC. Data are normalized to ß-actin mRNA levels (^*^p < 0.05 vs. chow); *n* = 7. All data represent mean values ± SEM (^*^p < 0.05). Conditioned medium (CM), High fat diet (HFD).

Animals under HFD gained more weight compared to animals given a chow diet (Figure 1A). To monitor the impact of obesity on PAR2 expression in the vasculature of these mice, we analyzed PAR2 levels in aortas carefully controlled to be free of adipose tissue. *PAR2* mRNA expression in aortas from HFD-fed animals was significantly higher (ΔΔCt of 1.2 ± 0.1) than in chow diet-fed animals (ΔΔCt of 0.8 ± 0.1) (Figure 1B). Furthermore, *PAR2* expression was positively correlated with the body weight of corresponding animals (Figure 1C). To determine whether the observed changes in aortic *PAR2* expression were specifically related to AT, we analyzed the effect of CM from murine adipose tissue explants on HCSMC. Exposure of HCSMC to CM of animals under chow diet had no effect on *PAR2* content while treatment with CM obtained from HFD-fed animals provoked a 2-fold increase (Figure 1D).

**Figure 2:**
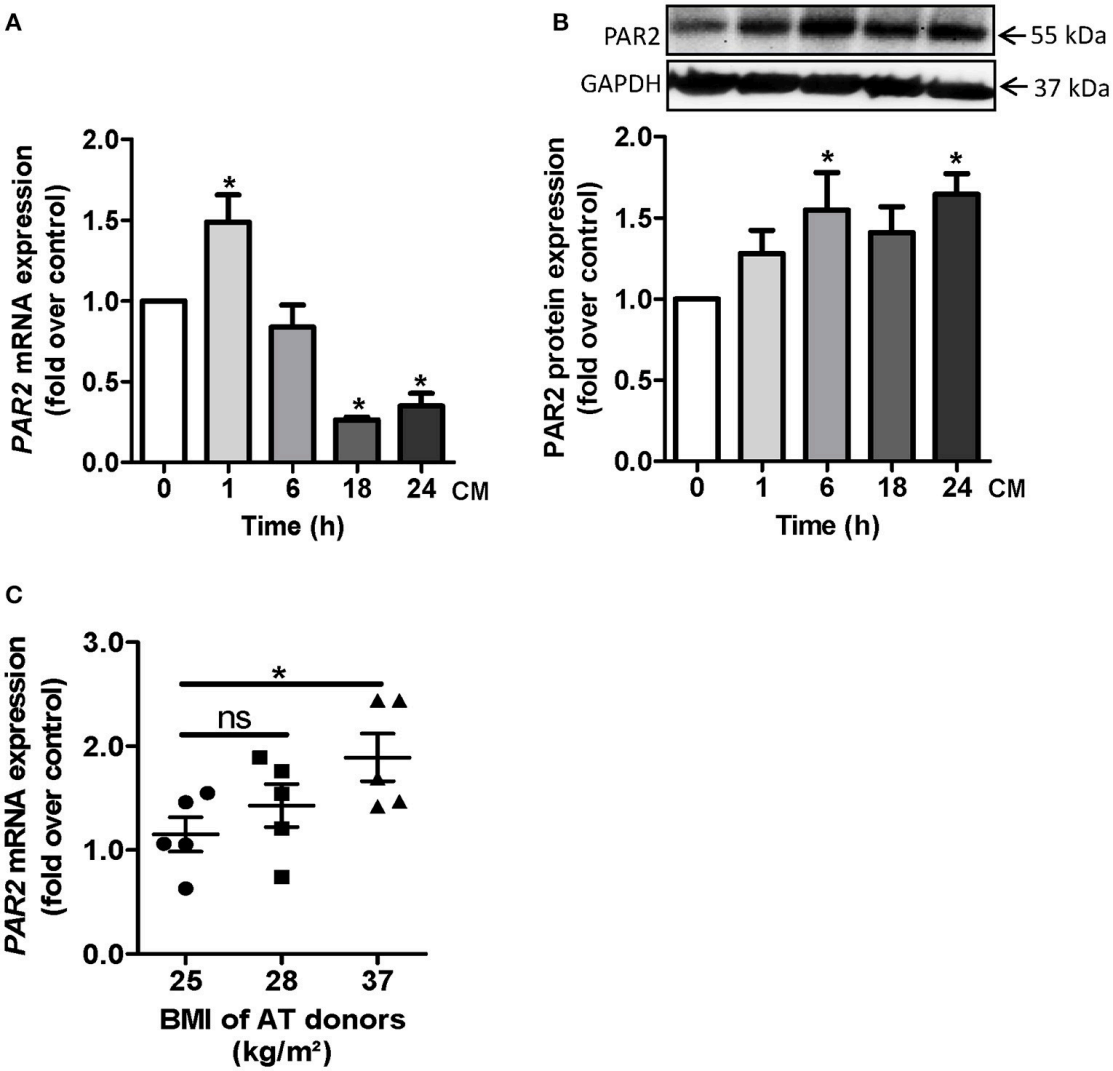
**PAR2 induction by adipocyte-derived factors in HCSMC**. **(A,B)** Time course of PAR2 mRNA and protein expression after CM treatment for indicated time points was assessed by qRT-PCR and western blot in HCSMC. Data were normalized to β-actin or GAPDH levels respectively; *n* = 4–6 (^*^p < 0.05 vs. time 0). **(C)** PAR2 expression in HCSMC after challenge to CM for 1 h and its relation to BMI of AT donors, *n* = 15 (^*^p < 0.05). Data represent mean values ± SEM. Conditioned medium (CM), human coronary smooth muscle cells (HCSMC).

Certain cytokines, which are elevated in obesity such as TNF-α or IL-1α are able to induce PAR2 (Nystedt et al., 1996; Hamilton et al., 2001). In order to explore the impact of adipokines on PAR2 induction, we generated CM from differentiated primary human adipocytes obtained from overweight or obese subjects (BMI 30.1 ± 1.9 kg/m^2^). Human vascular cells were exposed to adipocyte CM. In HCSMC, *PAR2* mRNA was significantly elevated up to 1.5 ± 0.2 fold over control after 1 h CM treatment (Figure 2A). At protein level an increase in PAR2 expression occurred after 6 and 24 h (1.7 ± 0.2 fold over control, respectively; Figure 2B). Moreover, *PAR2* expression was enhanced in HCSMC exposed to CM of obese subjects. While CM of subjects with a BMI of 25 kg/m^2^ was only capable to induce *PAR2* 1.2 ± 0.2 fold compared to non-treated cells, CM of subjects showing a BMI of 37 kg/m^2^ could induce *PAR2* to a significantly higher extent (Figure 2C).

### PAR2 mediates CM-induced proliferation in HCSMC

**Figure 3:**
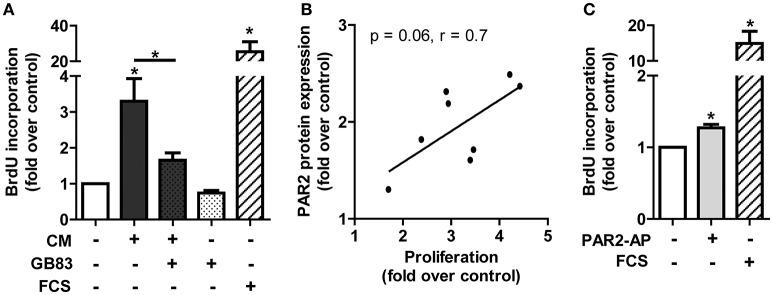
**PAR2 mediated proliferation in HCSMC**. **(A)** Proliferation was assessed by BrdU incorporation after 1 h pre-incubation with GB83 (10 μM) and subsequent treatment with either CM alone or in combination with GB83 for 24 h in HCSMC. FCS (5%) was included as a positive control. **(B)** Correlation of PAR2 induction after 24 h and proliferation rate by CM; *n* = 8. **(C)** Effects of PAR2-AP (50 μM) on proliferation was analyzed after 24 h; *n* = 4–6. Data represent mean values ± SEM (^*^p < 0.05 vs. control). Conditioned medium (CM), fetal calf serum (FCS), human coronary smooth muscle cells (HCSMC), PAR2-activating peptide (PAR2-AP).

A change in intima-media thickness is an important event in the development of vascular remodeling. (Langille, 1993) During this process proliferation of smooth muscle cells results in a thickening of the *tunica media* (Langille, 1993). Therefore, using CM from human adipocytes we assessed proliferation in HCSMC. Treatment of HCSMC with CM increased proliferation 3.3 ± 0.6 fold over control. Interestingly, we observed that this effect was PAR2-dependent, since it was reduced by 70% with the PAR2 specific antagonist GB83 (Figure 3A). We further observed a trend toward higher mitogenic activity due to stronger PAR2 expression in HCSMC (Figure 3B, *p* = 0.06). PAR2 involvement during HCSMC proliferation was supported by the initiation of proliferation with PAR2-AP (Figure 3C).

### CM induced PAR2 levels and apoptosis in HCAEC

In HCAEC, CM treatment resulted in a biphasic increase of *PAR2* expression at mRNA level (Supplementary Figure 1A), whereas PAR2 was gradually enhanced at protein level with a peak of 2.1 ± 0.4 fold over control after 24 h (Supplementary Figure 1B). Since the proliferation of smooth muscle cells during vascular remodeling is accompanied by endothelial cell apoptosis (Langille, 1993), we investigated the impact of adipocyte CM on caspase3/7 activity in HCAEC. Apoptosis of HCAEC was increased by CM and effects of CM were prevented by the PAR2 antagonist GB83 (Supplementary Figure 1C). GB83 alone had no effect on either cell proliferation or apoptosis.

### Induction of cyclooxygenase 2 levels occurs via PAR2

**Figure 4:**
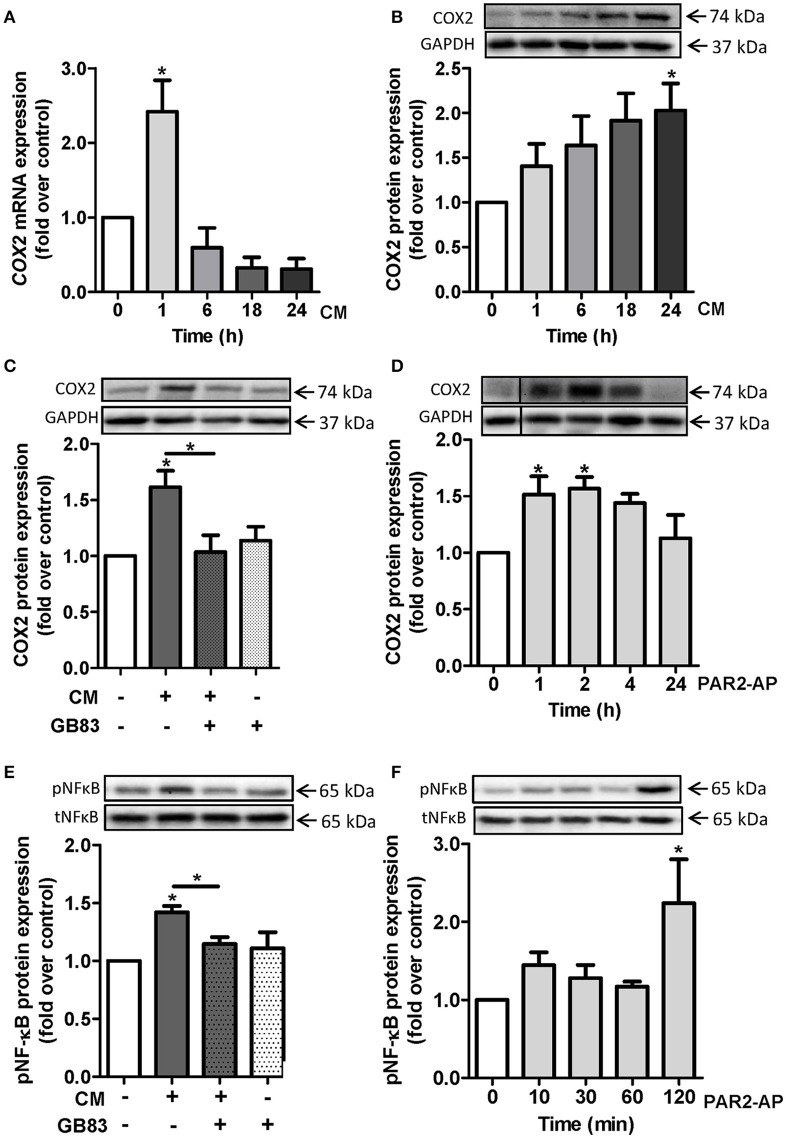
**CM induces COX2 expression in HCSMC via PAR2**. COX2 expression was determined in HCSMC exposed to CM for the indicated time points and **(A)** mRNA and **(B)** protein levels were quantified. *n* = 4–6. **(C)** CM-induced COX2 expression after 24 h and pre-incubation with PAR2 antagonist GB83 (10 μM); *n* = 6. **(D)** Time course of COX2 induction by PAR2-AP (50 μM); *n* = 6. **(E)** CM-induced pNF-κB expression after 24 h and pre-incubation with PAR2 antagonist GB83, *n* = 4. **(F)** Time course of pNF-κB expression by PAR2-AP, *n* = 5. Data of mRNA target gene levels are normalized to β-actin. Protein expression of COX was normalized to GAPDH and pNF-κB was normalized to tNF-κB. Data are mean values ± SEM (^*^p < 0.05 vs. time 0 or control). Conditioned medium (CM), human coronary smooth muscle cells (HCSMC), PAR2-activating peptide (PAR2-AP).

Since cyclooxygenase 2 (COX2) is a key mediator of inflammation and vascular dysfunction (Vane and Botting, 1998; Bagi et al., 2006), we investigated the potential regulation of COX2 by CM. We exposed HCSMC cells to CM for the indicated time points. CM treatment triggered an enhancement of *COX2* expression at mRNA level with a peak at 1 h (2.4 ± 0.4 fold over control; Figure 4A) and a peak at protein levels at 24 h (2 ± 0.3 fold over control; Figure 4B). One hour pre-incubation of HCSMC with the specific PAR2 antagonist GB83 downregulated CM-induced COX2 expression to basal levels (Figure 4C). Additionally, we detected a rise in COX2 protein level after challenging cells with the PAR2-AP for 1 h (1.5 ± 0.2 fold over control) and 2 h (1.6 ± 0.1 fold over control; Figure 4D). As a marker of pro-inflammatory signaling, we examined phosphorylation of the transcription factor NF-κB. After 24 h CM-induced phosphorylation of NF-κB was prevented by GB83 (Figure 4E). Accordingly, phosphorylation of NF-κB in HCSMC was induced by the PAR2 agonist PAR2-AP (Figure 4F).

### PAR2 activation leads to proliferation and COX2 induction in a VEGFR2 dependent-manner

**Figure 5:**
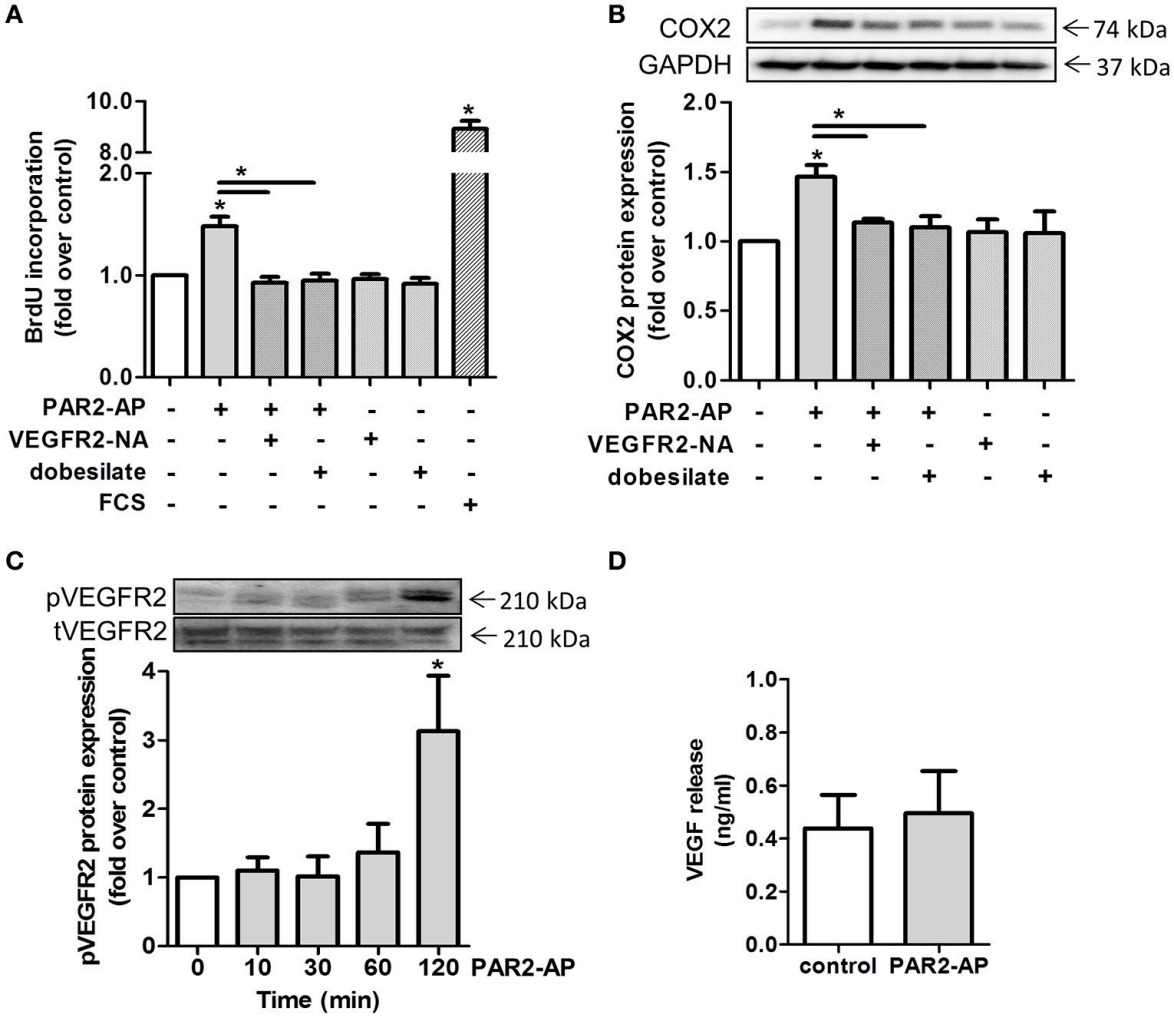
**PAR2 mediated proliferation and COX2 upregulation requires intracellular VEGFR2 transactivation. (A)** PAR2-AP-induced proliferation. HCSMC were pre-treated with dobesilate (100 μM) or VEGFR2-NA (50 ng/ml) for 1 h, *n* = 4. **(B)** Effects of dobesilate and VEGFR2-NA on COX2 protein expression after 2 h; *n* = 3. **(C)** Time course of the PAR2-AP-mediated phosphorylation of the VEGFR2 (Tyr1059); *n* = 4. **(D)** PAR2-AP-triggered VEGF release from HCSMC after 24 h, *n* = 7. Data are mean values ± SEM (^*^p < 0.05 vs. time 0). Fetal calf serum (FCS), PAR2-activating peptide (PAR2-AP), VEGFR2 neutralizing antibody (VEGFR2-NA).

As previously described by Schlich et al., VEGF which signals through the vascular endothelial growth factor receptor 2 (VEGFR2) (Ferrara et al., 2003), is a main contributor to smooth muscle cell proliferation (Schlich et al., 2013). Here, we found increased VEGF levels in CM of adipose explants from mice upon HFD (Supplementary Figure 2A). In addition, VEGF release from human adipocytes correlated with BMI of adipose tissue explant donors (Supplementary Figure 2B). In order to explore if the VEGFR2 was involved in PAR2-mediated proliferation and COX2 induction, we blocked the VEGFR2 by usage of dobesilate and VEGFR2-NA. Indeed, pre-incubation with either the inhibitor dobesilate or a VEGFR2-NA for 1 h and subsequent treatment with PAR2-AP resulted in a reduction of proliferation back to basal levels (1.0 ± 0.1 fold and to 0.9 ± 0.1 fold compared to control levels, respectively). VEGFR2 blockade alone had no effect on HCSMC proliferation (Figure 5A). Furthermore, PAR2-AP-mediated COX2 induction of 1.5 ± 0.1 fold over control was diminished to 1.1 ± 0.03 fold over control after VEGFR2-NA and to 1.1 ± 0.1 fold over control after dobesilate addition (Figure 5B). Next, we explored if PAR2 activation affected VEGFR2 phosphorylation. After 2 h we observed an enhanced receptor phosphorylation (Figure 5C), while total VEGFR2 content was unaltered (Data not shown). However, VEGFR2 activation did not occur due to increased VEGF levels since exposure of HCSMC to PAR2-AP did not alter VEGF release compared to control conditions (Figure 5D).

## Discussion

The development of obesity is associated with functional and structural changes in the AT (Ouchi et al., 2011). Particularly, during obesity, AT switches to a more pro-inflammatory secretome (Ouchi et al., 2011). These secreted factors, upregulated in obesity, reach the vascular wall through the circulation where they can exert deleterious effects (Goldstein and Scalia, 2007). As a consequence, induction of vascular inflammation, endothelial dysfunction and smooth muscle cell proliferation takes place. These processes are hallmarks of vascular diseases and have been linked to PAR2 (Hirano and Kanaide, 2003; Wronkowitz et al., 2014; Romacho et al., 2016). To our knowledge, there are scarce reports about a direct connection between obesity and PAR2 in the vasculature. In the present study we aimed to explore the effect of CM as a model of circulating adipokines under obesogenic conditions. We show that obesity caused *PAR2* upregulation in murine aortas, and that CM of obese subjects triggered PAR2 induction in HCSMC. Furthermore, CM induced HCSMC proliferation, COX2 and pNF-κB in a PAR2-dependent manner. We demonstrated that PAR2 activation and subsequent effects involved a transactivation of the VEGFR2.

It has been reported that obesity upregulates PAR2 locally in human AT as well as in AT of rats fed a HFD (Lim et al., 2013). Adipokines released from AT are now acknowledged as mediators of inter-organ crosstalk between the AT and the vascular wall (Romacho et al., 2014). Indeed, pro-inflammatory adipokines, which are upregulated in obesity, such as IL-1α, IL-1β, and TNF-α have been described as potent PAR2 inducers in the vasculature (Nystedt et al., 1996; Hamilton et al., 2001; Ritchie et al., 2007). Moreover, in human aorta PAR2 expression is enhanced in atherosclerotic lesions (Napoli et al., 2004). Therefore, we aimed to explore if a low-grade chronic inflammatory condition such as obesity had an impact on PAR2 expression not only in AT itself (Lim et al., 2013), but also in the vascular wall. Interestingly, 24 weeks of HFD increased *PAR2* mRNA expression in murine aortas. *PAR2* expression in aortas was positively correlated with the body weight of mice. Furthermore, solely CM from animals undergoing HFD was able to induce *PAR2* in HCSMC. These findings were further corroborated in human vascular cells, namely HCAEC and HCSMC, where human adipocyte-derived CM promoted PAR2 upregulation on mRNA and protein levels. *PAR2* mRNA expression was positively correlated to the BMI of AT donors. Altogether these data suggest that an obesogenic environment provokes changes in the AT secretome leading to PAR2 upregulation in the vascular wall.

Vascular remodeling, characterized by proliferation and migration of smooth muscle cells as well as apoptosis of endothelial cells (Langille, 1993), is an early event in the development of atherosclerosis (Lerman et al., 1998). We recently demonstrated that adipocyte-derived CM can initiate HCSMC proliferation (Schlich et al., 2013). In addition we proved the participation of PAR2 in adipokine-induced HCSMC proliferation (Wronkowitz et al., 2014). Our data suggest that CM-mediated HCSMC proliferation as well as CM-mediated HCAEC apoptosis is PAR2 dependent since it is abolished by the PAR2 specific antagonist GB83 (Adams et al., 2012). The specificity of this antagonist has been previously demonstrated elsewhere (Barry et al., 2010). Accordingly, PAR2-AP induced HCSMC proliferation thereby confirming the involvement of PAR2. This result is in agreement with previous studies where PAR2 agonist induced proliferation of smooth muscle (Bono et al., 1997). Considering that PAR2 activation has been shown to result in migration of smooth muscle cells (Pena et al., 2012), these findings point toward a considerable role of PAR2 in vascular alterations. Further observations showed a trend for correlation between CM-induced HCSMC proliferation and PAR2 expression in these cells. Therefore, we assume that PAR2 abundance, upregulated by several CM-derived factors such as TNFα and IL-1β (Nystedt et al., 1996; Ritchie et al., 2007), might facilitate proliferation. However, the precise relevance of PAR2 induction in the context of vascular and metabolic diseases remains poorly understood and has to be further elucidated.

There is evidence for a pivotal role of PAR2 during atherosclerosis (Dery et al., 1998; Wronkowitz et al., 2014). It is known that PAR2 agonism can lead to NFκB activation in human coronary smooth muscle cells (Bretschneider et al., 1999; Wronkowitz et al., 2014). To further elucidate the role of PAR2 in this process, we explored the impact of PAR2 agonism both NFκB, as a master regulator of inflammatory gene expression (Macfarlane et al., 2001) and COX2, another key regulator of vascular inflammatory processes (Bagi et al., 2006). We found that CM activated NF-κB and induced COX2 via PAR2. Analogously, PAR2-AP significantly enhanced COX2 expression and phosphorylation of NF-κB in HCSMC. Consistently, the PAR2 antagonist GB83 prevented these deleterious effects highlighting the importance of this receptor during atherogenesis and inflammation-related diseases. Our results are in line with other studies which showed PAR2-AP-stimulated COX2 expression in HUVECs (Syeda et al., 2006) and activation of NF-κB in smooth muscle cells (Bretschneider et al., 1999). We therefore assume a critical role of PAR2 in controlling COX2- and NF-κB -mediated inflammation. However, one limitation of our study is that we have not dissected the precise interaction between COX2 and NF-κB.

Since it is known that the COX2 promoter region contains putative NFκB binding sites in smooth muscle cells (Tazawa et al., 1994; Nie et al., 2003) we can speculate that PAR2 activation most likely leads to NFκB translocation to the nucleus resulting in COX2 expression. Nontheless, COX2 products such as PGE_2_ are able to increase the transcription of NFκB, thus positively enhancing the inflammatory signaling (Poligone and Baldwin, 2001). So far there are no data available if and how exactly PAR2-mediated signaling pathways involve both NFκB and COX2. In the light of our data we cannot specifically dissect whether NFκB leads to COX2 induction, or if COX2 induction leads to NF-κB activity.

In this study we have focused on the effects of CM on inflammation and proliferation as two key processes in atherogenesis, since the role of PAR2 in adipokine-induced impairment of vascular reactivity (Romacho et al., 2016) and the effect of CM on endothelial migration has already been proven (Hu et al., 2013). CM contains numerous molecules derived from adipocytes, which serve as potential ligands for PAR2, thereby contributing to promote PAR2-mediated effects, such as proliferation. We have previously demonstrated that the adipokine DPP4 is an activator of PAR2 existing in the CM and driving proliferation through ERK1/2 activation in a PAR2-dependent manner (Wronkowitz et al., 2014). However, our group showed that CM-mediated HCSMC proliferation is mainly due to VEGF abundance (Schlich et al., 2013), which is even higher during obesity (Silha et al., 2005; Loebig et al., 2010; Disanzo and You, 2014). Among the potential factors present in the CM promoting PAR2-mediated proliferation and inflammation we now identified VEGF as an adipokine upregulated in murine CM from AT explants upon HFD (Supplementary Figure 2A). Moreover, VEGF abundance is positively correlated to BMI of human adipocyte donors (Supplementary Figure 2B). VEGF signaling occurs via receptor tyrosine kinases (RTKs) particularly VEGF receptor 1 and 2 (VEGFR1 and VEGFR2), of whom the latter is known to be responsible for most of the VEGF-induced actions, including proliferation (Waltenberger et al., 1994; Ferrara et al., 2003; Shibuya and Claesson-Welsh, 2006). In the present study, we prove that PAR2 is involved in HCSMC proliferation. Therefore, we hypothesize that PAR2 and VEGFR2 interaction may lead to COX2 induction resulting in HCSMC proliferation. Nevertheless, there are several factors in the CM, which have to be identified in future studies, potentially leading to PAR2 activation, induction or both *via* distinct mechanisms such as binding to the binding pocket at the extracellular loop 2 (sDPP4) (Wronkowitz et al., 2014), the cleavage of the N-terminus and unmasking of the tethered ligand (trypsin, cathepsin S) or the disarming of the receptor (cathepsin G) (Kagota et al., 2016). Synthetic PAR2-AP activate PAR2 due to binding to the binding pocket located at the extracellular loop 2 (Adams et al., 2011) which might result in divergent effects compared with CM-mediated actions. Furthermore, we cannot discard the participation of other novel mediators of atherogenesis such as miRNAS (Blumensatt et al., 2014; Novak et al., 2015).

Receptor crosstalk between PARs, other GPCRs and RTKs such as the VEGF receptor has been observed in various cell types for example endothelial or smooth muscle cells (Gschwind et al., 2001; Chandrasekharan et al., 2010; Gieseler et al., 2013; Mazor et al., 2013). To determine if receptor interaction is required in PAR2-mediated proliferation and COX2 upregulation, we assessed the effect of the VEGFR2 inhibitor dobesilate (Angulo et al., 2011) and a VEGFR2-NA. Specific activation of PAR2 by addition of PAR2-AP induced proliferation and COX2 expression which were suppressed by both dobesilate and the VEGFR2-NA. Furthermore, activation of PAR2 resulted in phosphorylation of VEGFR2. Altogether, these results point toward a PAR2-induced transactivation of VEGFR2. Since both receptors are present on the surface of the same cell types, receptor interaction is plausible. Receptor crosstalk occurs via different components such as the release of agonists or transactivation due to intracellular signal transducers (Gieseler et al., 2013). To shed more light onto underlying mechanisms we analyzed whether transactivation of VEGFR2 was ligand dependent or not. The PAR2 agonist did not increase VEGF release from HCSMC indicating that VEGFR2 transactivation is VEGF independent. However, VEGF production in response to PAR2 activation has been described in other cell types (Liu and Mueller, 2006; Dutra-Oliveira et al., 2012; Rasmussen et al., 2012). Nonetheless, other intracellular molecules such as tyrosine kinase Src, ROS and protein tyrosine phosphatases have been discussed to be potential mediators of RTK and PAR interaction (Gieseler et al., 2013). In our case, preliminary data discard the participation of Src (data not shown). Data on receptor transactivation are based mainly on findings focusing on PAR1 and RTKs, others than VEGFR2, thereby demonstrating the novelty of our results. Since precise mechanisms whereby PAR2 promotes activation of VEGFR2 and subsequent proliferation as well as COX2 induction are not yet fully understood, only further investigations will shed light on the specific mechanisms mediating this novel PAR2-VEGFR2 transactivation.

## Conclusion

We have demonstrated a pivotal role of PAR2 in obesity-related pro-atherogenic events. Adipocyte-derived CM from obese subjects triggered PAR2 induction in HCAEC and HCSMC. In turn, PAR2 upregulation might facilitate CM-induced proliferation and COX2 induction in HCSMC. We propose that during obesity upregulation and activation of PAR2 by elevated levels of pro-inflammatory adipokines in the circulation, results in NFκB activation, COX-2 induction and proliferation, key processes in the development of atherosclerosis (GRAPHICAL ABSTRACT). Importantly, these pro-atherogenic events involved transactivation of the VEGFR2 by PAR2. Therefore, we propose an essential function of PAR2 in vascular cells and present PAR2 as a potentially useful therapeutic target in the treatment of obesity-associated atherogenesis. Therefore, it is of great importance to investigate the exact mechanisms by which PAR2 transactivates the VEGFR2 and to identify certain PAR2 activators triggering the observed effects.

## Author contributions

II: data collection and analysis, study design, drafted the manuscript. TR: study design, critical revision of the manuscript. JE: study design, critical revision of the manuscript. All authors read and approved the final manuscript.

## Funding

This work was supported by grants from the Ministerium für Wissenschaft und Forschung des Landes Nordrhein-Westfalen (Ministry of Science and Research of the State of North Rhine-Westphalia), the Bundesministerium für Gesundheit (Federal Ministry of Health). TR was the recipient of FP7 Marie Curie Intra-European fellowship (ADDIO-PIEF-2012-328793).

### Conflict of interest statement

The authors declare that the research was conducted in the absence of any commercial or financial relationships that could be construed as a potential conflict of interest.

## Abbreviations

AT: Adipose tissue
BMI: Body mass index
CM: Conditioned media
COX2: Cyclooxygenase 2
CVD: Cardiovascular disease
FCS: Fetal calf serum
GAPDH: Glyceraldehyde-3-phosphate dehydrogenase
HCAEC: Human coronary artery endothelial cells
HCSMC: Human coronary smooth muscle cells
HFD: High fat diet
PAR2: Protease-activated receptor 2
PAR2-AP: Protease-activated receptor 2 activating peptide
SEM: Standard error of the mean
VEGF: Vascular endothelial growth factor
VEGFR2: Vascular endothelial growth factor receptor 2
VEGFR2-NA: Vascular endothelial growth factor receptor 2 neutralizing antibody.

## References

[B1] Adams M. N. Pagel C. N. Mackie E. J. Hooper J. D. (2012). Evaluation of antibodies directed against human protease-activated receptor-2. Naunyn Schmiedebergs Arch. Pharmacol. 385, 861–873. 10.1007/s00210-012-0783-6 22842724

[B2] Adams M. N. Ramachandran R. Yau M. K. Suen J. Y. Fairlie D. P. Hollenberg M. D.. (2011). Structure, function and pathophysiology of protease activated receptors. Pharmacol. Ther. 130, 248–282. 10.1016/j.pharmthera.2011.01.003 21277892

[B3] Aman M. Hirano M. Kanaide H. Hirano K. (2010). Upregulation of proteinase-activated receptor-2 and increased response to trypsin in endothelial cells after exposure to oxidative stress in rat aortas. J. Vasc. Res. 47, 494–506. 10.1159/000313877 20431298

[B4] Angulo J. Peiro C. Romacho T. Fernandez A. Cuevas B. Gonzalez-Corrochano R.. (2011). Inhibition of vascular endothelial growth factor (VEGF)-induced endothelial proliferation, arterial relaxation, vascular permeability and angiogenesis by dobesilate. Eur. J. Pharmacol. 667, 153–159. 10.1016/j.ejphar.2011.06.015 21703259

[B5] Bagi Z. Erdei N. Papp Z. Edes I. Koller A. (2006). Up-regulation of vascular cyclooxygenase-2 in diabetes mellitus. Pharmacol. Rep. 58(Suppl), 52–56. 17332672

[B6] Barry G. D. Suen J. Y. Le G. T. Cotterell A. Reid R. C. Fairlie D. P. (2010). Novel agonists and antagonists for human protease activated receptor 2. J. Med. Chem. 53, 7428–7440. 10.1021/jm100984y 20873792

[B7] Blumensatt M. Wronkowitz N. Wiza C. Cramer A. Mueller H. Rabelink M. J.. (2014). Adipocyte-derived factors impair insulin signaling in differentiated human vascular smooth muscle cells via the upregulation of miR-143. Biochim. Biophys. Acta 1842, 275–283. 10.1016/j.bbadis.2013.12.001 24333576

[B8] Bono F. Lamarche I. Herbert J. M. (1997). Induction of vascular smooth muscle cell growth by selective activation of the proteinase activated receptor-2 (PAR-2). Biochem. Biophys. Res. Commun. 241, 762–764. 10.1006/bbrc.1997.7847 9434782

[B9] Bretschneider E. Kaufmann R. Braun M. Wittpoth M. Glusa E. Nowak G.. (1999). Evidence for proteinase-activated receptor-2 (PAR-2)-mediated mitogenesis in coronary artery smooth muscle cells. Br. J. Pharmacol. 126, 1735–1740. 10.1038/sj.bjp.0702509 10372815 PMC1565962

[B10] Chandrasekharan U. M. Waitkus M. Kinney C. M. Walters-Stewart A. DiCorleto P. E. (2010). Synergistic induction of mitogen-activated protein kinase phosphatase-1 by thrombin and epidermal growth factor requires vascular endothelial growth factor receptor-2. Arterioscler. Thromb. Vasc. Biol. 30, 1983–1989. 10.1161/ATVBAHA.110.212399 20671228 PMC2956164

[B11] Cicala C. Pinto A. Bucci M. Sorrentino R. Walker B. Harriot P.. (1999). Protease-activated receptor-2 involvement in hypotension in normal and endotoxemic rats *in vivo*. Circulation 99, 2590–2597. 10.1161/01.CIR.99.19.2590 10330393

[B12] Dery O. Corvera C. U. Steinhoff M. Bunnett N. W. (1998). Proteinase-activated receptors: novel mechanisms of signaling by serine proteases. Am. J. Physiol. 274(6 Pt 1), C1429–C1452. 9696685 10.1152/ajpcell.1998.274.6.C1429

[B13] Despres J. P. (2012). Body fat distribution and risk of cardiovascular disease: an update. Circulation 126, 1301–1313. 10.1161/CIRCULATIONAHA.111.067264 22949540

[B14] Disanzo B. L. You T. (2014). Effects of exercise training on indicators of adipose tissue angiogenesis and hypoxia in obese rats. Metabolism 63, 452–455. 10.1016/j.metabol.2013.12.004 24412283

[B15] Dutra-Oliveira A. Monteiro R. Q. Mariano-Oliveira A. (2012). Protease-activated receptor-2 (PAR2) mediates VEGF production through the ERK1/2 pathway in human glioblastoma cell lines. Biochem. Biophys. Res. Commun. 421, 221–227. 10.1016/j.bbrc.2012.03.140 22497886

[B16] Emilsson K. Wahlestedt C. Sun M. K. Nystedt S. Owman C. Sundelin J. (1997). Vascular effects of proteinase-activated receptor 2 agonist peptide. J. Vasc. Res. 34, 267–272. 10.1159/000159233 9256086

[B17] Ferrara N. Gerber H. P. LeCouter J. (2003). The biology of VEGF and its receptors. Nat. Med. 9, 669–676. 10.1038/nm0603-669 12778165

[B18] Gieseler F. Ungefroren H. Settmacher U. Hollenberg M. D. Kaufmann R. (2013). Proteinase-activated receptors (PARs) - focus on receptor-receptor-interactions and their physiological and pathophysiological impact. Cell Commun. Signal. 11:86. 10.1186/1478-811X-11-86 24215724 PMC3842752

[B19] Goldstein B. J. Scalia R. (2007). Adipokines and vascular disease in diabetes. Curr. Diab. Rep. 7, 25–33. 10.1007/s11892-007-0006-6 17254515

[B20] Greenberg A. S. Obin M. S. (2006). Obesity and the role of adipose tissue in inflammation and metabolism. Am. J. Clin. Nutr. 83, 461S–465S. 16470013 10.1093/ajcn/83.2.461S

[B21] Gschwind A. Zwick E. Prenzel N. Leserer M. Ullrich A. (2001). Cell communication networks: epidermal growth factor receptor transactivation as the paradigm for interreceptor signal transmission. Oncogene 20, 1594–1600. 10.1038/sj.onc.1204192 11313906

[B22] Hamilton J. R. Frauman A. G. Cocks T. M. (2001). Increased expression of protease-activated receptor-2 (PAR2) and PAR4 in human coronary artery by inflammatory stimuli unveils endothelium-dependent relaxations to PAR2 and PAR4 agonists. Circ. Res. 89, 92–98. 10.1161/hh1301.092661 11440983

[B23] Hauner H. Petruschke T. Russ M. Rohrig K. Eckel J. (1995). Effects of tumour necrosis factor alpha (TNFα) on glucose transport and lipid metabolism of newly-differentiated human fat cells in cell culture. Diabetologia 38, 764–771. 10.1007/s001250050350 7556976

[B24] Hill J. O. Wyatt H. R. Peters J. C. (2012). Energy balance and obesity. Circulation 126, 126–132. 10.1161/CIRCULATIONAHA.111.087213 22753534 PMC3401553

[B25] Hirano K. Kanaide H. (2003). Role of protease-activated receptors in the vascular system. J. Atheroscler. Thromb. 10, 211–225. 10.5551/jat.10.211 14566084

[B26] Hotamisligil G. S. Arner P. Caro J. F. Atkinson R. L. Spiegelman B. M. (1995). Increased adipose tissue expression of tumor necrosis factor-alpha in human obesity and insulin resistance. J. Clin. Invest. 95, 2409–2415. 10.1172/JCI117936 7738205 PMC295872

[B27] Hu L. Zhao J. Liu J. Gong N. Chen L. (2013). Effects of adipose stem cell-conditioned medium on the migration of vascular endothelial cells, fibroblasts and keratinocytes. Exp. Ther. Med. 5, 701–706. 10.3892/etm.2013.887 23403954 PMC3570169

[B28] Kagota S. Maruyama K. McGuire J. J. (2016). Characterization and functions of protease-activated receptor 2 in obesity, diabetes, and metabolic syndrome: a systematic review. Biomed. Res. Int. 2016:3130496. 10.1155/2016/3130496 27006943 PMC4781943

[B29] Karastergiou K. Mohamed-Ali V. (2010). The autocrine and paracrine roles of adipokines. Mol. Cell Endocrinol. 318, 69–78. 10.1016/j.mce.2009.11.011 19948207

[B30] Lamers D. Schlich R. Greulich S. Sasson S. Sell H. Eckel J. (2011). Oleic acid and adipokines synergize in inducing proliferation and inflammatory signalling in human vascular smooth muscle cells. J. Cell Mol. Med. 15, 1177–1188. 10.1111/j.1582-4934.2010.01099.x 20518853 PMC3822630

[B31] Langille B. L. (1993). Remodeling of developing and mature arteries: endothelium, smooth muscle, and matrix. J. Cardiovasc. Pharmacol. 21(Suppl. 1), S11–S17. 10.1097/00005344-199321001-00003 7681126

[B32] Lerman A. Cannan C. R. Higano S. H. Nishimura R. A. Holmes D. R. Jr. (1998). Coronary vascular remodeling in association with endothelial dysfunction. Am. J. Cardiol. 81, 1105–1109. 10.1016/S0002-9149(98)00135-0 9605050

[B33] Lim J. Iyer A. Liu L. Suen J. Y. Lohman R. J. Seow V.. (2013). Diet-induced obesity, adipose inflammation, and metabolic dysfunction correlating with PAR2 expression are attenuated by PAR2 antagonism. FASEB J. 27, 4757–4767. 10.1096/fj.13-232702 23964081

[B34] Liu Y. Mueller B. M. (2006). Protease-activated receptor-2 regulates vascular endothelial growth factor expression in MDA-MB-231 cells via MAPK pathways. Biochem. Biophys. Res. Commun. 344, 1263–1270. 10.1016/j.bbrc.2006.04.005 16650817

[B35] Lloyd-Jones D. Adams R. J. Brown T. M. Carnethon M. Dai S. De S. G.. (2010). Heart disease and stroke statistics–2010 update: a report from the American Heart Association. Circulation 121, e46–e215. 10.1161/CIRCULATIONAHA.109.192667 20019324

[B36] Loebig M. Klement J. Schmoller A. Betz S. Heuck N. Schweiger U.. (2010). Evidence for a relationship between VEGF and BMI independent of insulin sensitivity by glucose clamp procedure in a homogenous group healthy young men. PLoS ONE 5:e12610. 10.1371/journal.pone.0012610 20830305 PMC2935379

[B37] Macfarlane S. R. Seatter M. J. Kanke T. Hunter G. D. Plevin R. (2001). Proteinase-activated receptors. Pharmacol. Rev. 53, 245–282. 11356985

[B38] Mazor R. Alsaigh T. Shaked H. Altshuler A. E. Pocock E. S. Kistler E. B.. (2013). Matrix metalloproteinase-1-mediated up-regulation of vascular endothelial growth factor-2 in endothelial cells. J. Biol. Chem. 288, 598–607. 10.1074/jbc.M112.417451 23155052 PMC3537058

[B39] McGuire J. J. (2004). Proteinase-activated Receptor 2 (PAR2): a challenging new target for treatment of vascular diseases. Curr. Pharm. Des. 10, 2769–2778. 10.2174/1381612043383656 15320742

[B40] Mirza H. Yatsula V. Bahou W. F. (1996). The proteinase activated receptor-2 (PAR-2) mediates mitogenic responses in human vascular endothelial cells. J. Clin. Invest. 97, 1705–1714. 10.1172/JCI118597 8601636 PMC507235

[B41] Molino M. Raghunath P. N. Kuo A. Ahuja M. Hoxie J. A. Brass L. F.. (1998). Differential expression of functional protease-activated receptor-2 (PAR-2) in human vascular smooth muscle cells. Arterioscler. Thromb. Vasc. Biol. 18, 825–832. 10.1161/01.ATV.18.5.825 9598843

[B42] Morrish N. J. Wang S. L. Stevens L. K. Fuller J. H. Keen H. (2001). Mortality and causes of death in the WHO multinational study of vascular disease in diabetes. Diabetologia 2, S14–S21. 10.1007/PL00002934 11587045

[B43] Napoli C. de N. F. Wallace J. L. Hollenberg M. D. Tajana G. De R.G.. (2004). Evidence that protease activated receptor 2 expression is enhanced in human coronary atherosclerotic lesions. J. Clin. Pathol. 57, 513–516. 10.1136/jcp.2003.015156 15113859 PMC1770305

[B44] Nie M. Pang L. Inoue H. Knox A. J. (2003). Transcriptional regulation of cyclooxygenase 2 by bradykinin and interleukin-1beta in human airway smooth muscle cells: involvement of different promoter elements, transcription factors, and histone h4 acetylation. Mol. Cell. Biol. 23, 9233–9244. 10.1128/MCB.23.24.9233-9244.2003 14645533 PMC309638

[B45] Novak J. Olejnickova V. Tkacova N. Santulli G. (2015). Mechanistic role of microRNAs in coupling lipid metabolism and atherosclerosis. Adv. Exp. Med. Biol. 887, 79–100. 10.1007/978-3-319-22380-3_5 26662987 PMC4871243

[B46] Nystedt S. Emilsson K. Wahlestedt C. Sundelin J. (1994). Molecular cloning of a potential proteinase activated receptor. Proc. Natl. Acad. Sci. U.S.A. 91, 9208–9212. 10.1073/pnas.91.20.9208 7937743 PMC44781

[B47] Nystedt S. Ramakrishnan V. Sundelin J. (1996). The proteinase-activated receptor 2 is induced by inflammatory mediators in human endothelial cells. Comparison with the thrombin receptor. J. Biol. Chem. 271, 14910–14915. 10.1074/jbc.271.25.14910 8663011

[B48] Ouchi N. Parker J. L. Lugus J. J. Walsh K. (2011). Adipokines in inflammation and metabolic disease. Nat. Rev. Immunol. 11, 85–97. 10.1038/nri2921 21252989 PMC3518031

[B49] Pena E. Arderiu G. Badimon L. (2012). Subcellular localization of tissue factor and human coronary artery smooth muscle cell migration. J. Thromb. Haemost. 10, 2373–2382. 10.1111/j.1538-7836.2012.04910.x 22938499

[B50] Poligone B. Baldwin A. S. (2001). Positive and negative regulation of NF-κB by COX-2: roles of different prostaglandins. J. Biol. Chem. 276, 38658–38664. 10.1074/jbc.M106599200 11509575

[B51] Rasmussen J. G. Riis S. E. Frobert O. Yang S. Kastrup J. Zachar V.. (2012). Activation of protease-activated receptor 2 induces VEGF independently of HIF-1. PLoS ONE 7:e46087. 10.1371/journal.pone.0046087 23049945 PMC3457954

[B52] Ritchie E. Saka M. Mackenzie C. Drummond R. Wheeler-Jones C. Kanke T.. (2007). Cytokine upregulation of proteinase-activated-receptors 2 and 4 expression mediated by p38 MAP kinase and inhibitory κB kinase β in human endothelial cells. Br. J. Pharmacol. 150, 1044–1054. 10.1038/sj.bjp.0707150 17339845 PMC2013917

[B53] Romacho T. Elsen M. Rohrborn D. Eckel J. (2014). Adipose tissue and its role in organ crosstalk. Acta Physiol. (Oxf). 210, 733–753. 10.1111/apha.12246 24495317

[B54] Romacho T. Vallejo S. Villalobos L. A. Wronkowitz N. Indrakusuma I. Sell H.. (2016). Soluble dipeptidyl peptidase-4 induces microvascular endothelial dysfunction through proteinase-activated receptor-2 and thromboxane A2 release. J. Hypertens. 34, 869–876. 10.1097/HJH.0000000000000886 26895560

[B55] Schlich R. Willems M. Greulich S. Ruppe F. Knoefel W. T. Ouwens D. M.. (2013). VEGF in the crosstalk between human adipocytes and smooth muscle cells: depot-specific release from visceral and perivascular adipose tissue. Mediat. Inflamm. 2013:982458. 10.1155/2013/982458 23935253 PMC3723083

[B56] Shibuya M. Claesson-Welsh L. (2006). Signal transduction by VEGF receptors in regulation of angiogenesis and lymphangiogenesis. Exp. Cell Res. 312, 549–560. 10.1016/j.yexcr.2005.11.012 16336962

[B57] Silha J. V. Krsek M. Sucharda P. Murphy L. J. (2005). Angiogenic factors are elevated in overweight and obese individuals. Int. J. Obes. (Lond) 29, 1308–1314. 10.1038/sj.ijo.0802987 15953938

[B58] Sriwai W. Mahavadi S. Al-Shboul O. Grider J. R. Murthy K. S. (2013). Distinctive G protein-dependent signaling by protease-activated receptor 2 (PAR2) in smooth muscle: feedback inhibition of RhoA by cAMP-independent PKA. PLoS ONE 8:e66743. 10.1371/journal.pone.0066743 23825105 PMC3688948

[B59] Syeda F. Grosjean J. Houliston R. A. Keogh R. J. Carter T. D. Paleolog E.. (2006). Cyclooxygenase-2 induction and prostacyclin release by protease-activated receptors in endothelial cells require cooperation between mitogen-activated protein kinase and NF-κB pathways. J. Biol. Chem. 281, 11792–11804. 10.1074/jbc.M509292200 16467309

[B60] Taube A. Schlich R. Sell H. Eckardt K. Eckel J. (2012). Inflammation and metabolic dysfunction: links to cardiovascular diseases. Am. J. Physiol. Heart Circ. Physiol. 302, H2148–H2165. 10.1152/ajpheart.00907.2011 22447947

[B61] Tazawa R. Xu X. M. Wu K. K. Wang L. H. (1994). Characterization of the genomic structure, chromosomal location and promoter of human prostaglandin H synthase-2 gene. Biochem. Biophys. Res. Commun. 203, 190–199. 10.1006/bbrc.1994.2167 8074655

[B62] Trayhurn P. Drevon C. A. Eckel J. (2011). Secreted proteins from adipose tissue and skeletal muscle - adipokines, myokines and adipose/muscle cross-talk. Arch. Physiol. Biochem. 117, 47–56. 10.3109/13813455.2010.535835 21158485

[B63] Vane J. R. Botting R. M. (1998). Anti-inflammatory drugs and their mechanism of action. Inflamm. Res. 47(Suppl. 2), S78–S87. 10.1007/s000110050284 9831328

[B64] Waltenberger J. Claesson-Welsh L. Siegbahn A. Shibuya M. Heldin C. H. (1994). Different signal transduction properties of KDR and Flt1, two receptors for vascular endothelial growth factor. J. Biol. Chem. 269, 26988–26995. 7929439

[B65] Wronkowitz N. Gorgens S. W. Romacho T. Villalobos L. A. Sanchez-Ferrer C. F. Peiro C.. (2014). Soluble DPP4 induces inflammation and proliferation of human smooth muscle cells via protease-activated receptor 2. Biochim. Biophys. Acta 1842, 1613–1621. 10.1016/j.bbadis.2014.06.004 24928308

[B66] Zhao P. Metcalf M. Bunnett N. W. (2014). Biased signaling of protease-activated receptors. Front. Endocrinol. (Lausanne) 5:67. 10.3389/fendo.2014.00067 24860547 PMC4026716

