# Supplementary material for: Protease-Activated Receptor 2 Promotes Pro-Atherogenic Effects through Transactivation of the VEGF Receptor 2 in Human Vascular Smooth Muscle Cells
Source: Front Pharmacol. 2017 Jan 4;7:497. doi: 10.3389/fphar.2016.00497 (PMC5209375; doi:10.3389/fphar.2016.00497)
Supplement: Supplementary file 1 [file DataSheet1.DOCX]

Supplementary Material

**Protease-Activated Receptor 2 Promotes Pro-Atherogenic Effects through Transactivation of the VEGF Receptor 2 in Human Vascular Smooth Muscle Cells.**

**Ira Indrakusuma1, Tania Romacho^1^*, Jürgen Eckel1,^2^***

^1^ Paul-Langerhans-Group for Integrative Physiology, German Diabetes Center, Düsseldorf, Germany

^2^ German Center for Diabetes Research (DZD e.V.), Düsseldorf, Germany

*both authors jointly directed this work

*** Corresponding author:**

Prof. Dr. Jürgen Eckel

Mail: Eckel@uni-duesseldorf.de

**Supplementary Figure 1: CM induces PAR2 and enhances apoptosis in HCAEC.** **(A-B)** CM induced PAR2 mRNA and protein expression in HCAEC over time. Data were normalized to β-actin or GAPDH, respectively; n=4-6 (*p<0.05 vs. time 0). **(C)** Caspase 3/7 activity in HCAEC exposed to CM for 18 hours and in combination with GB83 (10μM). 200 μM H2O2 was used as a positive control; n=5. Data are mean values ± SEM (*p<0.05 vs. control). Conditioned medium (CM), human coronary artery endothelial cells (HCAEC).

**Supplementary Figure 2: Secretion of adipokines from murine adipose explants or human adipocytes. (A)** Secretion profile of murine adipose tissue explants from chow- and HFD-fed animals; n=3-7 (*p<0.05). **(B)** Correlation of donor BMI and VEGF concentration in CM from human primary adipocytes from respective autopsy donors; n=9 Data are mean values ± SEM (*p<0.05). Body mass index (BMI), Conditioned medium (CM), vascular endothelial growth factor (VEGF).
