# Supplementary figures and images for: Protease-Activated Receptor 2 Promotes Pro-Atherogenic Effects through Transactivation of the VEGF Receptor 2 in Human Vascular Smooth Muscle Cells
Source: Front Pharmacol. 2017 Jan 4;7:497. doi: 10.3389/fphar.2016.00497 (PMC5209375; doi:10.3389/fphar.2016.00497)

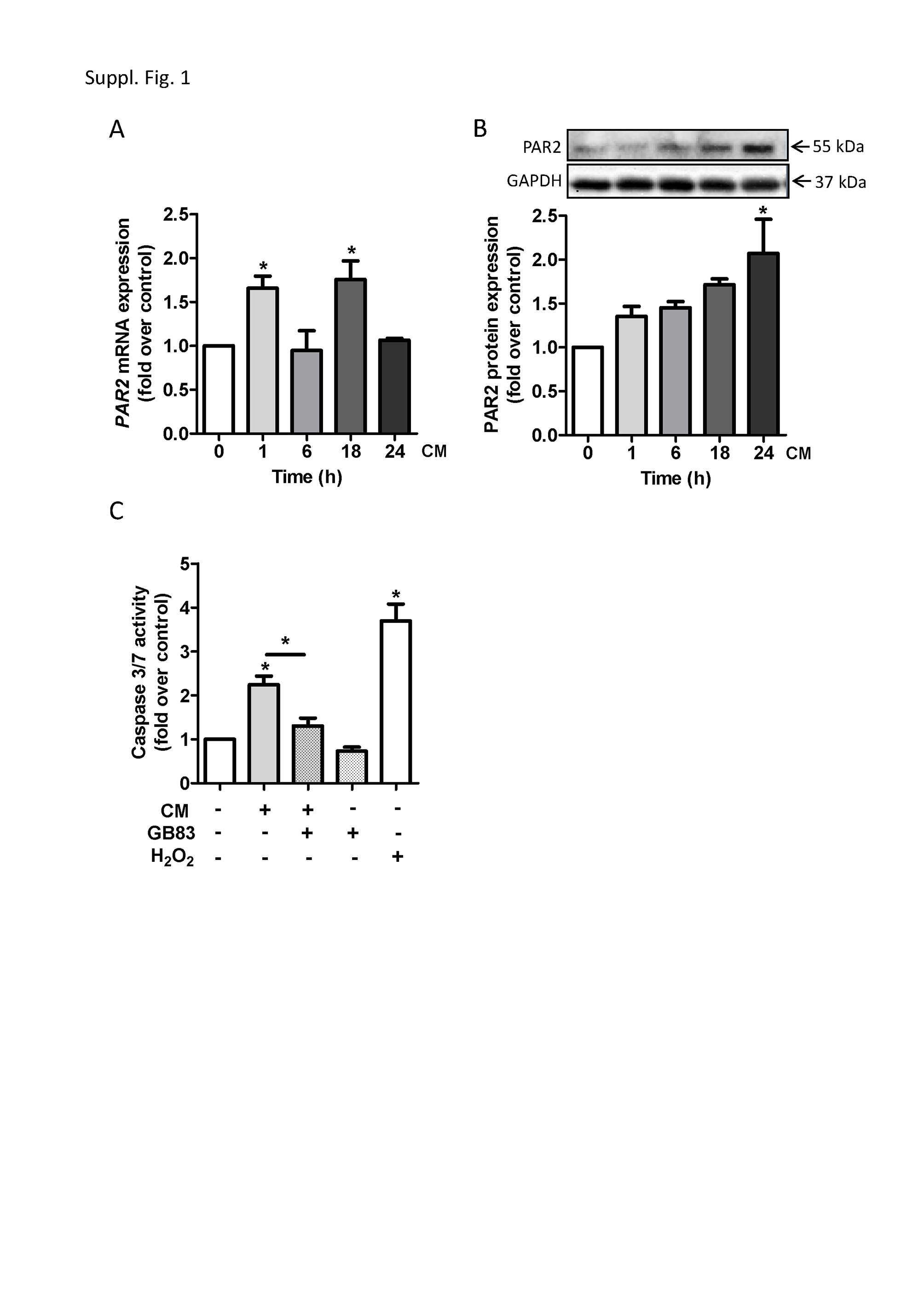

Supplement: Supplementary file 2 [file Image1.TIFF]

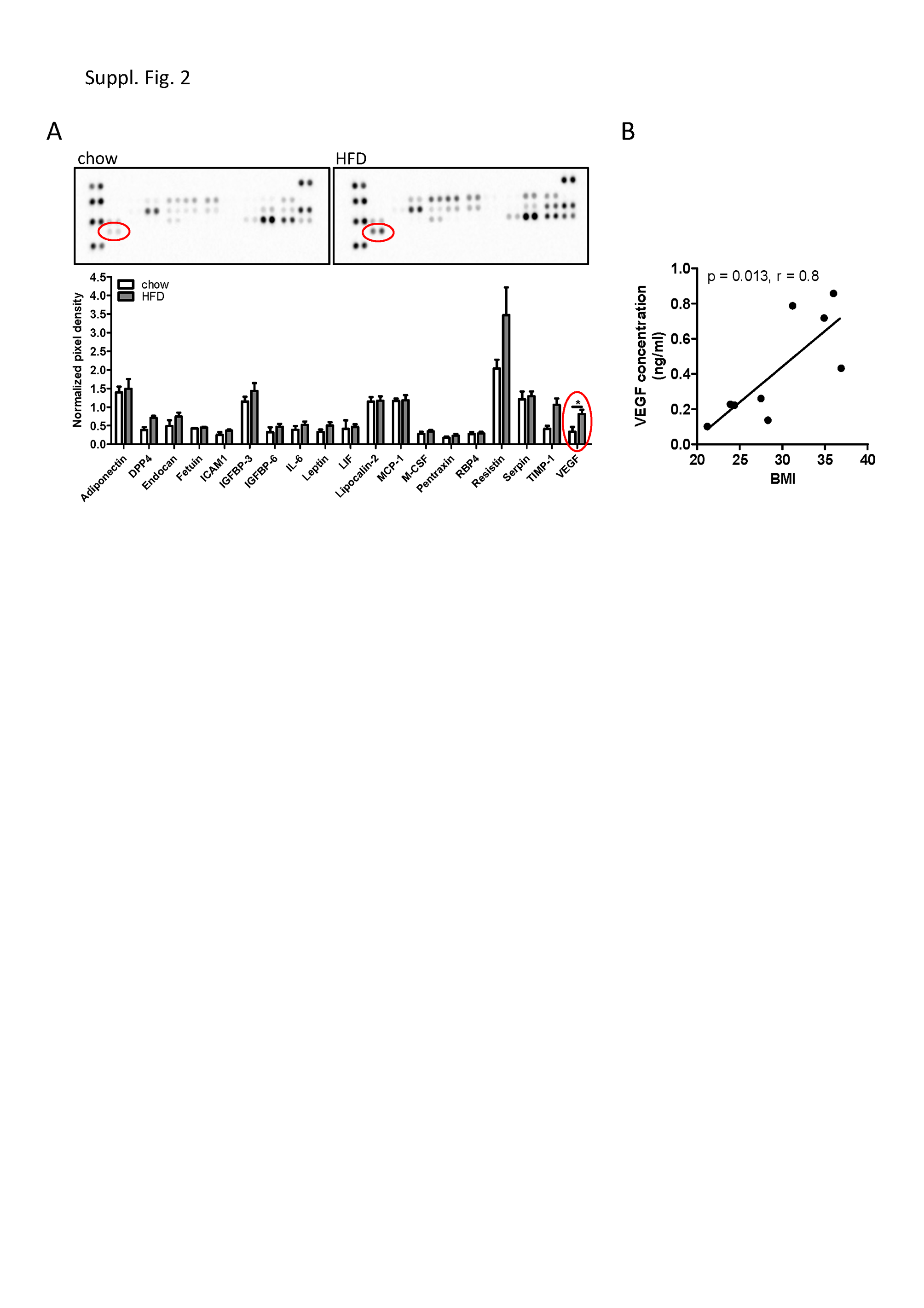

Supplement: Supplementary file 3 [file Image2.TIFF]
